# Seroprevalence of 16 *Leptospira* Serovars in Wild Boar (*Sus scrofa*) Hunted in Saxony-Anhalt, Germany

**DOI:** 10.3390/ani15182725

**Published:** 2025-09-18

**Authors:** Alice Stagnoli, Robert Valerio House, Juliane Hagemann, Karen Dohmann, Martin Pfeffer, Catrin Albrecht

**Affiliations:** 1 Department of Veterinary Medicine, State Office for Consumer Protection Saxony-Anhalt, 39576 Stendal, Germanycatrin.albrecht@sachsen-anhalt.de (C.A.); 2 Centre of Veterinary Public Health, Institute of Animal Hygiene and Veterinary Public Health, University of Leipzig, 04103 Leipzig, Germany

**Keywords:** *Leptospira*, wild boar, prevalence, *Leptospira fainei*, Saxony-Anhalt

## Abstract

In order to investigate the occurrence and epidemiology of leptospirosis in the wild boar (*Sus scrofa*) population of Saxony-Anhalt, Germany, blood samples were collected from 2616 wild boar regularly hunted in 2023 and 2024. The diagnostic investigations were performed using the microscopic agglutination test (MAT) using a panel of fifteen pathogenic and one intermediate serovar of *Leptospira* spp. Overall, 12.4% (325/2616) (CI 95% 11.2–13.7) of wild boar were seropositive, with Australis being the most prevalent serovar (22.8%), followed by Pomona (13.2%) and Pyrogenes (12.3%). Specific antibodies against the intermediate *Leptospira fainei* serovar Hurstbridge were also detected in 8% of the positive samples. Based on these results, we conclude that wild boar are regularly infected with different zoonotic *Leptospira* serovars. As these hunted animals are tested for other notifiable diseases, additional screening for specific antibodies against *Leptospira* spp. could be easily implemented. This would be an important tool for detecting human and domestic animals’ risk of exposure and may provide insight into which *Leptospira* serovars might be currently of clinical relevance.

## Simple Summary

Wild boar (*Sus scrofa*) are reservoirs for various pathogens, including *Leptospira* spp., posing a risk of infection to humans, domestic animals, and livestock. Between 2023 and 2024, a total of 2616 blood samples from regularly hunted wild boar in Saxony-Anhalt were tested for specific antibodies against 15 pathogenic and one intermediate *Leptospira* serovars using the microscopic agglutination test (MAT). In total, 12.4% (CI 95% 11.2–13.7) of the samples were positive for at least one *Leptospira* serovar. Most of the positive *Leptospira* results were for the serovar Australis (22.8%). Specific antibodies against the intermediate serovar Hurstbridge were also detected, which is considered the first detection in Germany. This study discusses the role of the wild boar in the epidemiology of leptospirosis and its implications for public health.

## 1. Introduction

Leptospirosis is one of the most common zoonoses worldwide, with 1.03 million estimated cases each year [1], posing a significant risk to both public and animal health. In Germany, leptospirosis in humans usually occurs as a sporadic disease. Between 2001 and 2024, an average of 110 human cases per year were reported [2].

However, as the symptoms of leptospirosis are often nonspecific and most cases have a subclinical or mild course, a high number of unreported cases is assumed.

Of great importance for the spread of leptospires are latently infected carrier animals with persistent *Leptospira* infection, which host the pathogens in the renal tubules and excrete them via urine, thereby infecting other individuals directly or indirectly. Although the brown rat (*Rattus norvegicus*) is the most important source of human infection due to its synanthropic lifestyle, a large spectrum of wild and domestic animals can serve as reservoir hosts [3], among which wild boar (*Sus scrofa*) have been identified as an important reservoir of leptospirosis in many European countries [4,5,6,7,8,9,10].

The taxonomy of *Leptospira* spp. is complex. To date, 68 different *Leptospira* species with more than 300 serovars, grouped in 20 serogroups, are known [11,12].

The term serogroup is of taxonomic importance and defines groups with antigenically related serovars. However, identical serovars may belong to different *Leptospira* species [13]. While molecular diagnostic techniques and detailed sequence typing are used for the characterization of *Leptospira* strains and genotypes, the microscopic agglutination test (MAT) is the most important tool for the categorization of serovars, and is still the gold standard in routine diagnostics [14,15,16]. Most of the studies have been carried out in Europe on pathogenic serovars of *Leptospira*, while knowledge on intermediate serovars is limited. Recently, in Italy, Cilia et al. [17] detected intermediate *Leptospira* serovar DNA in 6% of wild boar kidneys examined, proving that wild boar can also serve as a reservoir for intermediate serovars. Intermediate *Leptospira* spp. can also be pathogenic under specific circumstances, as they have been reported to cause mild to severe infections in several human studies [18,19].

Saxony-Anhalt, a federal state in Germany, as well as other European countries, hosts a robust wild boar population, making it an important focal point for the surveillance of wildlife and parasitic, bacterial, and viral diseases [20,21,22,23,24,25,26].

However, little is known about the prevalence of leptospires in the wild boar population from Saxony-Anhalt, in particular about the prevalence of intermediate serovars of *Leptospira* spp.

Therefore, the aim of the present study is to determine the current serological prevalence of leptospirosis in Saxony-Anhalt, based on data obtained by testing blood samples from legally hunted wild boar in the years 2023 and 2024 for specific antibodies against 15 pathogenic *Leptospira* serovars and one intermediate *Leptospira* (*L. fainei* serovar Hurstbridge). The findings aim to provide a more nuanced understanding of the potential role of wild boar as carriers and reservoirs of these bacteria, posing a possible risk to humans, domestic animals, and livestock.

## 2. Materials and Methods

### 2.1. Sample Collection

Wild boar were chosen over other species for this study because of their potential role as a reservoir for *Leptospira*. Therefore, blood samples routinely sent by hunters to the Department of Veterinary Medicine in the State Office for Consumer Protection of Saxony-Anhalt as part of monitoring for classical swine fever (Schweinepest-Monitoring-Verordnung) [27] have been used for the current investigation.

Blood samples were collected directly by hunters during the 2023 and 2024 hunting seasons by cardiac puncture shortly after death. Hunters were provided by our institute with a standardized form, on which they recorded relevant data for each animal, including date of hunting, hunting ground, gender, and age, considering three age classes: young (under 12 months old), sub-adult (between 12 and 24 months), and adult (over 24 months). Age estimation was based on dental eruption patterns, a method taught in mandatory training courses for hunters, particularly for species such as wild boar. Hunters were equipped with official sampling kits provided by our institute. Each kit contained sterile tubes (KABEVETTE^®^ 4.9 mL for hematological analyses and treated test tubes for serum collection KABE Labortechnik GmbH, Nümbrecht-Elsenroth, Germany), collection instructions and materials to help maintain sample quality until delivery.

Upon arrival, all samples were centrifuged at 1000× *g* for 15 min using a Rotanta 460 benchtop centrifuge (Andreas Hettich GmbH, Tuttlingen, Germany) to obtain serum or plasma and subsequently stored at −20 °C until testing. Samples with an inadequate quantity or quality of the supernatant were not included in the study.

In total, blood samples were analyzed from 2616 wild boar of two hunting years. The number of samples collected was 555 in 2023 and 2061 in 2024 from all 14 counties of Saxony-Anhalt.

### 2.2. Microscopic Agglutination Test (MAT)

Specific anti-*Leptospira* antibodies were detected by MAT using a panel of 16 *Leptospira* serovars as reported in Table 1. MAT is widely regarded as the gold standard serological assay for detecting antibodies against *Leptospira* spp. in veterinary diagnostics due to its high specificity and ability to identify serovar-specific antibodies, which is essential for epidemiological surveillance. Considering the field conditions and the nature of collected samples (blood sera and plasma), MAT was selected as the most practical and informative method for this study.

Rabbit antisera were used as positive controls for each investigated serovar, while sterilized saline water was used as a negative control. All reference strains and positive controls were provided by the WOAH Reference Laboratory for Leptospirosis, Amsterdam University Medical Centre, Department of Medical Microbiology and Infection Prevention, Amsterdam, The Netherlands.

For strain maintenance, all employed strains were grown in Ellinghausen-MacCullough-Johnson-Harris (EMJH-Difco, Detroit, MI, USA) at 30 ± 2 °C for a minimum of 4 days and a maximum of 8 days according to the World Organisation for Animal Health (WOAH) Manual of Diagnostic Tests and Vaccines for Terrestrial Animals [15].

In order to standardize the test procedure, prior to starting the MAT, the density of the *Leptospira* suspension was adjusted to approximately 2 × 10^8^ leptospires per mL by estimating the number of leptospires per field by dark-field microscopy [15]. Both the density estimation and the evaluation of the tested samples were performed with an Axiostar Plus dark-field microscope (Carl-Zeiss, Jena, Germany).

Initially, all sera and plasma samples were screened for specific antibodies against the 16 *Leptospira* serovars using the MAT. Samples were first diluted 1:50, and an equal volume of each antigen suspension was added to each well of a 96-well microtiter plate, resulting in a final dilution of 1:100. Plates were incubated at 30 ± 2 °C for 2 h and subsequently examined by dark-field microscopy. A sample was considered positive if ≥50% of the *Leptospira* in the suspension were agglutinated. All positive samples were then subjected to endpoint titration using twofold serial dilutions ranging from 1:100 to 1:25,000 to determine the antibody titer. In cases of cross-reactivity (i.e., positive reaction to multiple serovars), the serovar showing the highest titer was considered the presumptive infecting serovar, following the recommendations of the WOAH Manual of Diagnostic Tests and Vaccines for Terrestrial Animals [15].

### 2.3. Statistical Analysis

Data were recorded and analyzed using Microsoft Office Excel (Microsoft Office LTSC Standard 2021). Additional statistical data analysis was performed using the R software packages rcompanion (Version 4.4.2). The chi-square test (Pearson’s chi-squared test with Yates’ continuity correction, X^2^) was performed to evaluate the *Leptospira* infection ratio in relation to sex (male or female), age class (young, sub-adult, or adult), and hunting year. Fisher’s exact test was used when the number of observations was equal to or less than five for the evaluation of the *Leptospira* serovar distribution among the counties.

Statistical analyses were conducted on two levels: first, the overall prevalence of specific anti-*Leptospira* antibodies in each district was compared to the overall prevalence of Saxony-Anhalt to identify significant differences. Second, the prevalence of individual *Leptospira* serovars within each district was compared to the average prevalence of the same serovar across Saxony-Anhalt to detect specific serovar clustering or deviations. A 95% confidence interval (CI) was used for all analyses, and the threshold for statistical significance was set at a *p*-value ≤ 0.05 [28].

## 3. Results

Of the total 2616 collected samples, 1014 (38.8%) were female and 1519 (58.1%) were male. The sex of 83 animals (3.2%) was not recorded; of these, 9 (10.8%) tested positive. In total, 111 females and 205 males tested positive for specific antibodies against leptospires, representing 10.9% and 13.5% of the examined animals, respectively.

**Figure 1:**
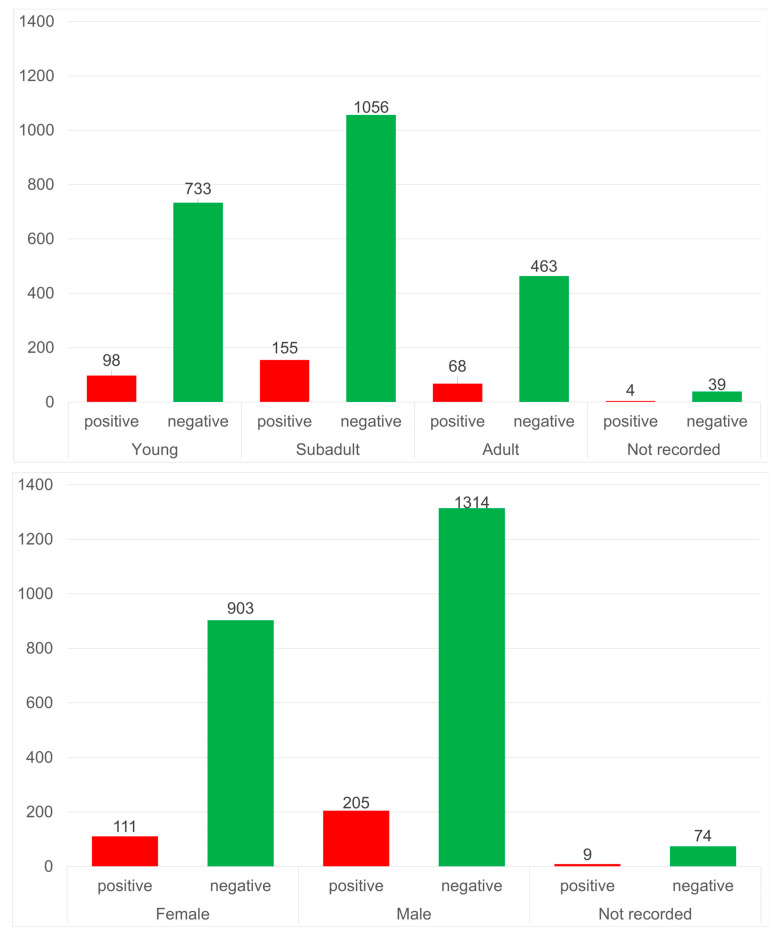
Total number of wild boar from Saxony-Anhalt examined in this study, hunted during 2023 and 2024, and stratified by age class (**upper panel**) and sex (**lower panel**).

Figure 1 provides a summary of the distribution of positive samples by sex and age group. A detailed breakdown of these results, including the number of animals sampled in 2023 and 2024 as well as their distribution across the different counties, is presented in the Supplementary Material (Table S1).

Considering the age of the examined wild boar, 831 (31.8%) were less than 1 year old, 1211 (46.3%) were between 1 and 2 years old, and 531 (20.3%) were over 2 years old. Age was not recorded for 43 (1.6%) animals. Specific antibodies against leptospires were found in all three age groups examined. The highest percentages of positive reactions were found in the groups of sub-adults and adults, with 155 (12.8%) and 68 (12.8%) positive samples, respectively. In the youngest age group, 98 (11.8%) positive samples were found. Taken together, no statistical differences were found for seropositive results in relation to hunting year, wild boar sex, or age class.

**Figure 2:**
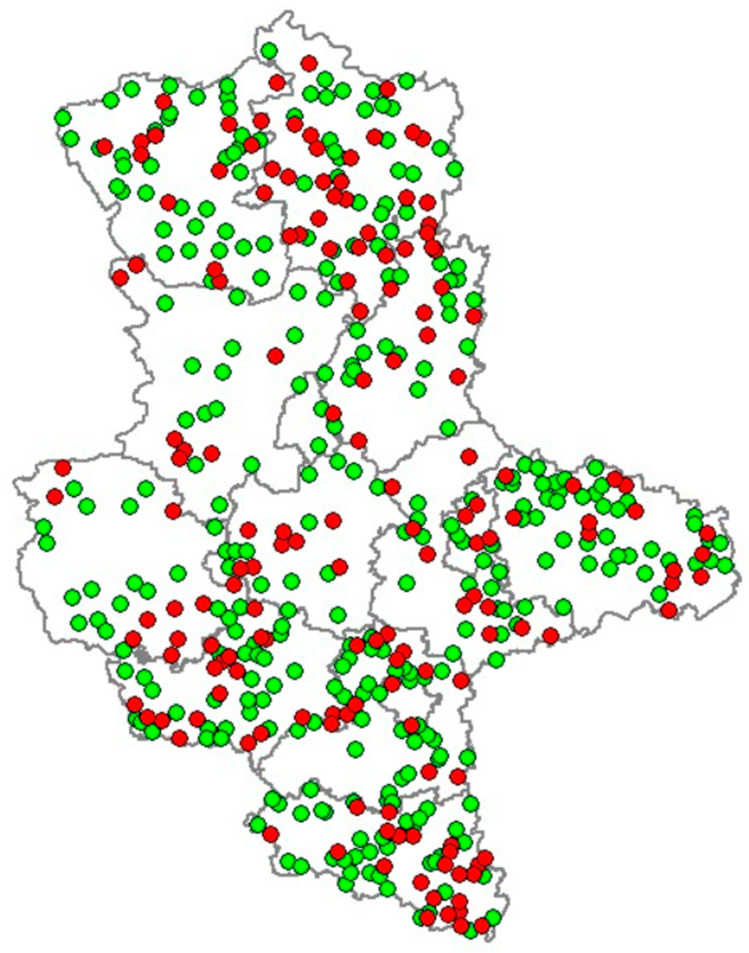
Distribution of positive (red dots) and negative (green dots) animals in the different counties of Saxony-Anhalt. Animals from the same location are represented by a single dot.

**Figure 3:**
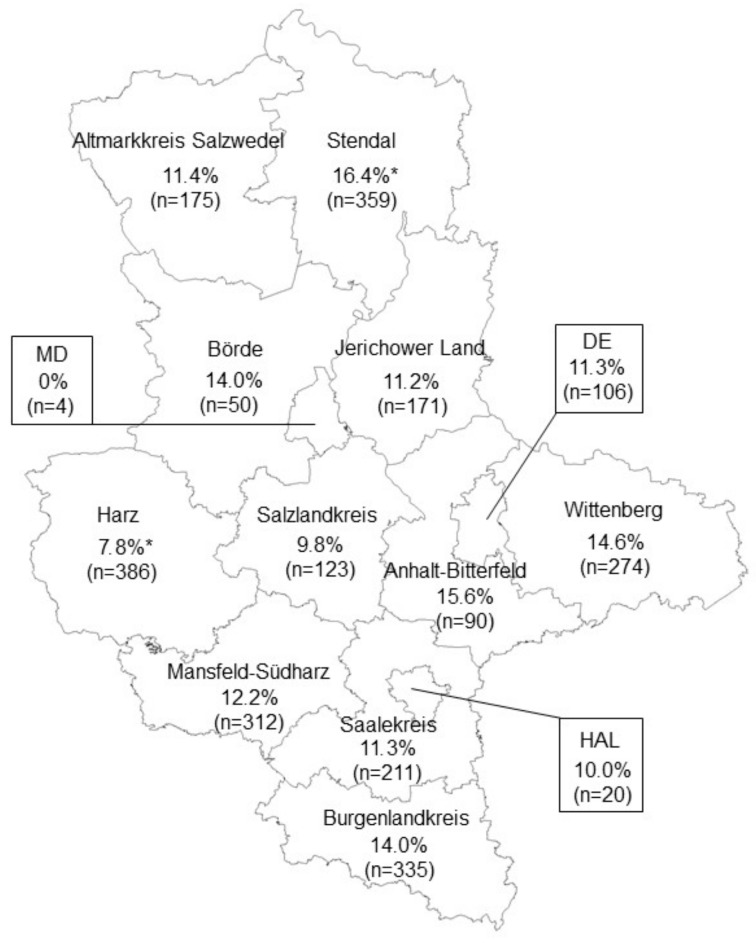
Apparent seroprevalence (%) of *Leptospira* in wild boar from the different administrative districts of Saxony-Anhalt: DE = Dessau-Roßlau, MD = Magdeburg city, HAL = Halle (Saale). Statistically significant differences in prevalence between districts are indicated by an asterisk (*).

Distribution of the positive and negative *Leptospira* cases in Saxony-Anhalt is shown in Figure 2, and the prevalence for each administrative district is represented in Figure 3.

With the exception of the district of Magdeburg city, *Leptospira* seropositive wild boar have been found in all administrative districts of Saxony-Anhalt. For the entire federal state, an apparent prevalence of 12.4% (CI 95% 11.2–13.7) has been calculated, including the results for all investigated specific anti-*Leptospira* antibodies. The highest number of positive reactions was detected in the district of Stendal with 16.4%, but other districts also had a wild boar population with a comparable prevalence of specific anti-*Leptospira* antibodies. Statistical significance was observed for the districts of Stendal and Harz (Figure 3).

Regarding the *Leptospira* serovar distribution, the serological results confirmed the presence of specific antibodies against thirteen pathogenic and one intermediate serovars. No seropositive reactions were found for the serovars Hardjo and Canicola.

**Figure 4:**
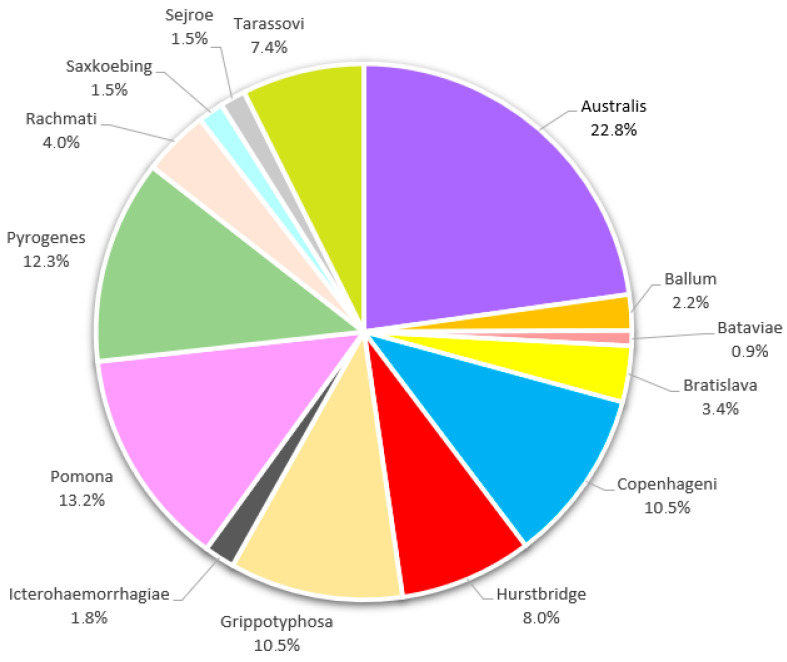
Distribution (%) of *Leptospira* serovars identified in wild boar (n = 325) positively tested samples by MAT. In animals with antibody reactivity to multiple serovars, only the one with the highest antibody titer was considered the presumptive infecting serovar and is therefore represented in the chart.

Specific antibodies were mainly directed against the serovars Australis (22.8%), Pomona (13.2%), and Pyrogenes (12.3%). The percentages for all detected serovars are reported in Figure 4.

The antibody titer of wild boar ranged from 1:100 to 1:1600. Most frequently, titers of 1:100 and 1:200 were detected. The highest titer, a dilution of 1:1600, was recorded against the serovar Australis (Table 2).

In order to evaluate a possibly increased presence of the investigated serovars, their prevalences in the administrative districts were compared with their total prevalences in Saxony-Anhalt. Of the 16 *Leptospira* serovars tested, the prevalence of four in a total of five districts was significantly higher than the average prevalence in Saxony-Anhalt, namely Copenhageni in the district of Saalekreis (*p* = 0.033), Hurstbridge in the district of Harz (*p* = 0.026), Icterohaemorrhagiae in the Altmarkkreis Salzwedel (*p* = 0.003), and Tarassovi in the district of Anhalt-Bitterfeld (*p* = 0.006) and the independent city of Dessau-Roßlau (*p* = 0.007).

## 4. Discussion

Understanding the factors contributing to the maintenance and transmission of leptospires in wildlife is essential for assessing the risk posed to both animal and public health. Among wildlife species, wild boar have attracted considerable attention due to their expanding populations and increasing interactions with human and domestic animal environments [20]. The ecology and behavior of wild boar contribute significantly to their role in the epidemiology of leptospirosis. Their frequent contact with moist environments through activities such as wallowing and foraging by burrowing in moist soil increases their exposure to *Leptospira* bacteria. This environmental interaction, combined with the potential for pathogen transmission through mucous membranes or skin lesions, underscores the importance of wild boar as key players in the maintenance and spread of leptospires. Supporting this, numerous serological surveys conducted worldwide have documented a high prevalence of specific antibodies against *Leptospira* spp. in wild boar populations [29,30].

The identified specific anti-*Leptospira* antibody prevalence of 12.4% in wild boar in our study is in the range of other European studies, e.g., in Poland (10.4%) [8], in Spain (14.6%) [31], in Italy (14.6%) [9], in the Czech Republic (16.9%) [32], in the federal state of Berlin, Germany (17.7%) [4], or in France (18.4%) [10]. Other studies in Europe have shown higher seroprevalences, e.g., in Croatia (26.0% and 31.9%) [6,33], in Slovenia, where the seroprevalence reached 45.5% [5], and in Portugal with 65.4% [34]. In contrast, clearly lower seroprevalences have been found in other studies, e.g., in Sweden (3.1%) [7] and also in Tuscany, Italy (6.0%) [35].

In addition to geographical and climatic differences, the data analysis and interpretation might be important for the study’s outcome. According to the WOAH Manual of Diagnostic Tests and Vaccines for Terrestrial Animals [15], in the case of a sample reactive to more than one serovar, we considered only the serovar with the highest antibody titer in the prevalence calculation. This applied to 64 wild boar in our dataset. Higher seroprevalence in other studies might also result from multiple counts of positive results against several *Leptospira* serovars in one sample. Furthermore, unlike other authors (e.g., [31]), in our study, only blood samples with an antibody titer of at least 1:100 were considered positive.

In our study, most antibody titers for the different serovars have been found between 1:100 and 1:200, a finding consistent with other serosurvey studies where low titers were frequently observed [5,32,34,35].

Findings regarding a correlation between anti-*Leptospira* antibodies and age are inconsistent. In line with Cilia et al., 2020 [9], we did not find an association between *Leptospira* exposure and age, as all age groups had almost identical seropositivity rates. Furthermore, we could not find a significant association between sex and seropositivity, comparable to other European countries (e.g., [36]).

As described by other authors [4,6,8,34,36], and also in our study, seroreactivity or cross-reactions of one sample to multiple serovars within a serogroup or different serogroup strains have been frequently observed.

Results regarding the predominant serovar(s) differ between the European studies. As in our study, Cilia et al. [9] and Roquelo et al. [10] also described Australis as the most represented serovar. In addition, the serovars Pomona and Copenhageni were also well represented in our study and in various other European studies. However, it should be noted that the serovar Australis has not been included in all published surveys.

Among the various *Leptospira* species identified, the presence of the intermediate serovar Hurstbridge is noteworthy. This species has been implicated in both human and veterinary infections [37,38,39,40], though it is less discussed in the context of European wildlife. To the best of the authors’ knowledge, the current study represents the first description of specific antibodies against serovar Hurstbridge in wild boar in Saxony-Anhalt as well as in the whole of Germany. Its identification in wild boar in Saxony-Anhalt may provide new insights into the potential of this pathogen to spread within the region’s wildlife populations. While the ecological role of intermediate serovar Hurstbridge remains to be fully understood, its detection in wild boar suggests that this species may play a previously underestimated role in the epidemiology of leptospirosis.

The seroprevalence of *Leptospira* in the wild boar population of Saxony-Anhalt provides important insight into the geographic and serovar-specific distribution of these pathogens. In particular, our study identified statistically significant differences in the presence of specific *Leptospira* serovars across the administrative districts of Saxony-Anhalt, such as *Leptospira* serovar Copenhageni in the Saalekreis, serovar Hurstbrige in the district Harz, serovar Ichterohaemorrhagiae in the district Altmarkkreis Salzwedel, and serovar Tarassovi in the districts Anhalt-Bitterfeld and the independent city Dessau-Roßlau. A significant clustering of specific serovars in certain districts suggests that environmental, ecological, and anthropogenic factors play a crucial role in the distribution of *Leptospira* infection in wild boar populations. Regional differences, encompassing both macroclimatic and microclimatic conditions, may influence the observed variation in leptospirosis seroprevalence.

Macroclimatic factors such as annual rainfall, mean temperature, and seasonal humidity, as well as microclimatic conditions including the presence of wetlands, forest cover, and soil moisture, can affect the environmental survival and transmission of *Leptospira* spp. These environmental variables, together with differences in habitat structure, land use, and host density, could lead to varying exposure risks for wild boar and, consequently, to the heterogeneous distribution of positive cases. Detailed investigation of these aspects was beyond the scope of the present study and will be addressed in future research. The wild boar itself is an opportunistic generalist occupying almost every habitat type within the state, so it may be challenging to decipher the factors responsible for the uneven serovar clustering.

A significant difference in seroprevalence was observed in some districts compared to the overall prevalence of Saxony-Anhalt, specifically for Harz and Stendal. These differences may be influenced by a combination of environmental and ecological factors, such as variation in habitat types, local climate conditions, and wild boar population densities, which can affect exposure risk to *Leptospira*. Additionally, differences in hunting practices and sample sizes between districts could also contribute to the observed variation. Further detailed investigation of these factors is needed to better understand the drivers behind the regional differences in seroprevalence.

Taken together, these findings underscore the need for targeted surveillance and preventive measures, particularly in areas where wild boar interact with livestock and other domestic species, to elucidate the precise ecological role of wild boar in the *Leptospira* transmission and to assess the potential risks to public health.

## 5. Conclusions

Based on the results of this investigation, wild boar might play an important role in the epidemiology of leptospirosis in Saxony-Anhalt. Of the 2616 wild boar tested, 325 (12.4%) showed specific antibodies against one or more *Leptospira* serovars. The seroprevalence found in this study is comparable to that reported in many other European countries. The three main serogroups circulating among wild boar in Saxony-Anhalt are Australis, Pomona, and Pyrogenes. The presence of specific antibodies against the intermediate serovar Hurstbridge in 8% of the examined samples should also be emphasized. These findings strengthen the need for further studies on this emerging serovar and to investigate a possible carrier role of wild boar and other species in the epidemiology of pathogenic and intermediate *Leptospira*. Furthermore, from a public health perspective, risk groups such as hunters, farmers, foresters, etc., should be aware of the risk of possible infection through contact with infected wild boar and take the necessary measures to avoid it.

## Figures and Tables

**Table 1:** Panel of 16 *Leptospira* serovars used by the microscopic agglutination test.

Species	Serovar	Serogroup	Strain
*Leptospira interrogans*	Australis	Australis	Ballico
	Bataviae	Bataviae	Swart
	Bratislava	Bratislava	Jez Bratislava
	Canicola	Canicola	Hond Utrecht IV
	Copenhageni	Icterohaemorrhagiae	M20
	Hardjo type Prajitno	Sejroe	Hardjoprajitno
	Icterohaemorrhagiae	Icterohaemorrhagiae	RGA
	Pomona	Pomona	Pomona
	Pyrogenes	Pyrogenes	Salinem
	Rachmati	Autumnalis	Rachmat
*Leptospira borgpetersenii*	Ballum	Ballum	MUS127
	Saxkoebing	Sejroe	MUS24
	Sejroe	Sejroe	M84
	Tarassovi	Tarassovi	Perepelitsin
*Leptospira kirschneri*	Grippotyphosa type Moskva	Grippotyphosa	Moskva V
*Leptospira fainei*	Hurstbridge	Hurstbridge	BUT6

**Table 2:** Distribution of *Leptospira* antibody titers for 325 positive wild boar hunted in 2023 and 2024 in Saxony-Anhalt.

*Leptospira* Serovar	Titer					Total (%)
	100	200	400	800	1600	
Australis	47	18	7	1	1	74 (22.8%)
Ballum	4	3	0	0	0	7 (2.2%)
Bataviae	3	0	0	0	0	3 (0.9%)
Bratislava	9	1	1	0	0	11 (3.4%)
Copenhageni	21	8	3	2	0	34 (10.5%)
Hurstbridge	20	3	2	1	0	26 (8.0%)
Grippotyphosa	29	4	1	0	0	34 (10.5%)
Icterohaemorrhagiae	4	1	1	0	0	6 (1.8%)
Pomona	32	9	1	1	0	43 (13.2%)
Pyrogenes	35	5	0	0	0	40 (12.3%)
Rachmati	11	2	0	0	0	13 (4.0%)
Saxkoebing	3	2	0	0	0	5 (1.5%)
Sejroe	4	0	1	0	0	5 (1.5%)
Tarassovi	16	7	1	0	0	24 (7.4%)

## Data Availability

The original data presented in the study are available upon request.

## Supplementary Materials

The following supporting information can be downloaded at https://www.mdpi.com/article/10.3390/ani15182725/s1: Table S1: Total number of examined wild boar from Saxony-Anhalt, hunted in 14 districts in 2023 (a) and 2024 (b). ABI: Anhalt-Bitterfeld, BK: Börde, BLK: Burgenlandkreis, DE: Dessau-Roßlau, HAL: Halle (Saale), HZ: Harz, JL: Jerichower Land, MD: Magdeburg-city, MSH: Mansfeld-Südharz, SK: Saalekreis, SLK: Salzlandkreis, SAW: Salzwedel, SDL: Stendal, WB: Wittenberg, ST. Saxony-Anhalt.
